# Effect of Antioxidants in the Treatment of COPD Patients: Scoping Review

**DOI:** 10.1155/2021/7463391

**Published:** 2021-11-24

**Authors:** Mauricio Orozco-Levi, Claudia Colmenares-Mejía, Jessica Ruíz, Yurley Dayanna Valencia-Barón, Alba Ramírez-Sarmiento, Doris Cristina Quintero-Lesmes, Norma C. Serrano

**Affiliations:** ^1^Servicio de Neumología, Fundación Cardiovascular de Colombia, Hospital Internacional de Colombia, Calle 155A No. 23-58 Floridablanca Santander/Valle de Menzuly Km 7 Piedecuesta, Santander, Colombia; ^2^Centro de Investigaciones Fundación Cardiovascular de Colombia, Calle 155A No. 23-58 Floridablanca, Santander, Colombia

## Abstract

Chronic obstructive pulmonary disease (COPD) is a common, preventable, treatable lung disease characterized by persistent respiratory symptoms and airflow limitation and multiorgan impact. This affects the nutritional status of patients and requires multidimensional interventions including nutritional interventions according to individual metabolic needs. Our scoping review determined the effects of antioxidants in the treatment of COPD patients and their role in the decrease in the probability of exacerbations, hospital readmissions, and changes in lung function. The sources MEDLINE, LILACS, and Google Scholar were consulted and 19 studies were selected. The most indicated antioxidants are N-Acetylcysteine, vitamins E and D, and Zinc. Other antioxidants from plants or fruits extracts are also being investigated. The beneficial effect of antioxidants in stable or exacerbated patients is not clear, but theoretical and biological arguments of benefit justify lines of research that specify the impact on reducing oxidative stress and negative effects in COPD.

## 1. Introduction 

Chronic obstructive pulmonary disease (COPD) is a common, preventable, and treatable disease characterized by persistent respiratory symptoms and airflow limitation that are due to abnormalities of the respiratory or alveolar tracts, generally caused by significant exposure to harmful particles or gases [1]. COPD is a group of progressive lung diseases. The most common of these diseases are emphysema and chronic bronchitis. Many people with COPD have both conditions. Emphysema slowly destroys air sacs in your lungs, which interferes with outward air flow. Bronchitis causes inflammation and narrowing of the bronchial tubes, which allows mucus to build up.

COPD imposes a high morbidity and mortality rate and is considered a first-line public health problem. According to data from the Global Burden of Disease Study, the prevalence of COPD in 2017 was 299.40 million cases worldwide [2]. The burden of COPD includes a mortality of 3.19 million cases (approximately 5% of all deaths in that year), which positioned it as the seventh cause of death among the 282 causes evaluated in the study [3].

The World Health Organization (WHO) estimates that, by 2030, COPD will be the third leading cause of death in the world, only behind ischemic heart disease and cerebrovascular disease (stroke), respectively. This would happen largely due to the increase in tobacco consumption [4]. Currently, the pharmacological treatment of patients with COPD focuses on the control of symptoms with the use of short- and long-acting bronchodilators or the combination of a long-acting beta-2 agonist bronchodilator and an inhaled corticosteroid; in addition, simultaneously with pharmacological treatment, it is recommended to use educational strategies on self-care habits, identification of symptoms, cessation of tobacco consumption, and vaccination against influenza, since they favor the quality of life of patients and reduce the rate of hospitalizations [5].

It has been documented that COPD not only affects the respiratory system but also involves other organs and systems, including the nutritional status of the patient [6], which significantly affects the evolution and prognosis of the disease. Therefore, the need for the use of interventions with approaches to respiratory therapy, physical exercise, psychological support, and nutritional support has gained strength in the recommendations within the management of patients with COPD [5]. Regarding this last intervention, it should be noted that its importance is due to the affectation of the nutritional status presented in people with chronic lung diseases, where it is estimated that between 10 and 45% of patients with COPD present malnutrition [7], especially after periods of exacerbation, where it is more accentuated. Nutritional alterations, both those with a low Body Mass Index (BMI) (<18.5 kg/m^2^) and overweight (>25 kg/m^2^) and obesity (>30 kg/m^2^), are common in COPD; however, the former is associated with higher mortality (RR = 1.34, 95% CI = 1.01–1.78), while overweight (RR = 0.47, 95% CI = 0.33–0.68) and obesity (RR = 0.59, 95% CI = 0.38–0.91) behave as protective factors for COPD, but being outside the normal weight ranges, they cause other comorbidities to appear [8].

**Figure 1:**
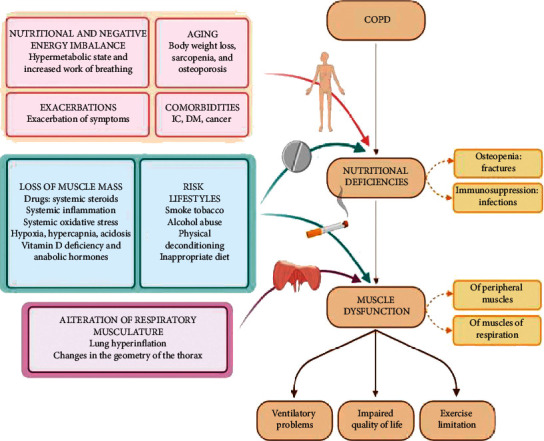
Factors that contribute to the presence of nutritional deficiency and muscle dysfunction in COPD patients (modified from Gea J Sancho-Muñoz A Chalela R Nutritional status and muscle dysfunction in chronic respiratory diseases: stable phase versus acute exacerbations. J Thorac Dis. 2018; 10 (12): S1332–S1354. doi:10.21037/jtd.2018.02.66; ^*∗*^ figure created on the BioRender platform).

Loss of muscle mass is one of the factors that contribute to the presence of nutritional deficiency in patients, favoring the appearance of muscle dysfunction (an extrapulmonary manifestation widely studied in COPD), and that includes the presence of abnormalities in strength and endurance of respiratory and peripheral muscles [9–11] caused by multisystemic factors with a deleterious effect, which increase the susceptibility of muscle fibers to membrane and sarcomere damage [12]. Both nutritional deficiency and muscle dysfunction are the result of the interaction of several factors, including smoking and physical deconditioning [13–15] (Figure 1). Interestingly, the lungs are highly susceptible to airborne pathogens and pollutants that mediate pathologies through generation of reactive oxygen species (ROS). One pathological consequence of excessive levels of ROS production is pulmonary diseases that account for large rates of mortality and morbidity in the world. Of the various mechanisms involved in pulmonary disease pathogenesis, mitochondrial dysfunction takes prominent importance. The significance of oxidative stress caused by ROS in pulmonary diseases suggests that antioxidants can represent complementary therapeutic strategies in COPD and other pulmonary diseases [16]. These factors are relevant because they increase ventilatory problems, limit exercise capacity, and deteriorate quality of life and patient survival [17]. It is extremely important to emphasize that skeletal muscle dysfunction occurs in patients with COPD and affects both ventilatory and nonventilatory muscle groups. It represents a very important comorbidity that is associated with poor quality of life and reduced survival. It results from a complex combination of functional, metabolic, and anatomical alterations leading to suboptimal muscle work. Muscle atrophy, altered fiber type and metabolism, and chest wall remodeling, in the case of the respiratory muscles, are relevant etiological contributors to this process. Muscle recovery measures consisting of a combination of pulmonary rehabilitation, optimized nutrition, and other strategies are associated with better prognosis when administered in stable patients as well as after that [18].

Bearing in mind the above, it is important to promote interventions with the use of nutritional supplements based both on the food and diet of the different populations and on the individual metabolic needs of the patients. It should be noted that patients with COPD and muscle loss have low concentrations of Branched Chain Amino Acids (BCAA), so it would be beneficial to include them in nutritional supplements, since their administration has been shown to improve muscle metabolism in patients [19]. Recently, different types of interventions have been studied, where the strategy with the greatest impact has been the use of antioxidants as an additional option to the usual treatment of COPD. “Antioxidant” is a term used to describe compounds that block lipid peroxidation and other oxidative reactions. Dietary antioxidants and antioxidant supplements have the potential to decrease reactive oxygen species (ROS) and prevent various chronic diseases [20].

Some of the most frequently used antioxidants are the following:

- Zinc (essential trace element): It is a chemical element that in the adult individual ranges between 1 and 2.5 g, with higher concentrations found in the liver, pancreas, kidneys, bones, and voluntary muscles. At the level of the respiratory system, it modulates the oxidative stress that is generated during the inflammatory response. The administration of Zinc at therapeutic doses (greater than the recommended daily intake) improves the infectious picture of respiratory diseases [21].
- Vitamin E: It is a powerful antioxidant micronutrient present in various foods that humans consume routinely. It provides protection to cells from free radicals, which are compounds that are generated after the conversion of ingested food into “energy.” Furthermore, vitamin E deficiency in COPD patients is associated with a greater fall in Forced Expiratory Volume (FEV1) throughout follow-up [22].
- Vitamin D: It is known as a hormone because its synthesis and main source of production in humans are when exposed to ultraviolet rays; in addition, it can be found in less quantity in some foods. Vitamin D deficiency is very common in COPD, occurring in around 60% of patients and its proportion is increased in subgroups where the disease is more severe [23]. However, its role in the disease is still not well understood. In vitro studies have shown that it is involved in inflammation and remodeling of the airways [22].
- Erdosteine: It is an original molecule derived from a natural amino acid, homocysteine in its N-thiolactonic form. The maximum concentration is reached within 1.2 hours after a single oral dose. In COPD, it lyses the glycoproteins that form mucus, causing ciliary transport and exerting antioxidant properties due to the action of the thionic groups released on free radicals [24].
- N-Acetylcysteine: It is a drug that has been tested the most in COPD patients. Its mucolytic effect generates positive results in the treatment of acute and chronic bronchial conditions. Furthermore, because it is an antioxidant, it complies with anti-inflammatory properties detected through the identification of a sulfhydryl group in its structure, which is oxidized and gives rise to disulfide bridges, allowing two molecules of the drug to be linked. These complexes, in the same way as reduced glutathione, proceed thanks to the reduction of radicals such as hydrogen peroxide, hydroxyl anion, and hypochlorous acid. While the inhibitory action of N-Acetylcysteine on molecular oxygen is almost nil, the reduction of hydroxyl anions is rapid and effective [25].
- Nutraceuticals: The European Nutraceutical Association (ENA) defines them as nutritional products that have relevant health effects, that are not synthetic substances or chemical compounds formulated for specific indications, and that contain nutrients (partially or in concentrated form) [26]. In COPD patients, the available scientific evidence indicates that some foods and nutrients, especially those nutraceuticals endowed with antioxidant and anti-inflammatory properties, when consumed in combinations and in the form of balanced dietary patterns, are associated with better lung function and even a decreased risk of disease [27].

Taking into account the variety of antioxidant options available, the objective of this scoping review was to determine the effect of the use of antioxidants in the treatment of COPD patients and the decrease in the likelihood of exacerbations, hospital readmissions, and lung function parameters.

## 2. Materials and Methods

A scoping review was developed. This type of review aims to identify the nature and scope of a future clinical research [28].

### 2.1. Information Sources and Literature Search

Three databases were consulted: MEDLINE via PubMed, LILACS, and Google Scholar. The search strategy was developed after identifying keywords (MeSH terms and free text in English and Spanish) based on the research question. The key terms used for the search were the following: (((((“Bronchitis, Chronic” [Mesh]) OR “Pulmonary Disease, Chronic Obstructive” [Mesh]) OR “Lung Diseases, Obstructive” [Mesh]) AND (“Therapeutics “[Mesh] OR” therapy “[Subheading])) AND (“Antioxidants “[Mesh] OR” Antioxidants “[Pharmacological Action])) OR” Dietary Supplements ”[Mesh] AND (exacerbations OR readmission OR lung function parameters). Additionally, study type filters were applied to focus the search on studies evaluating the effectiveness of the treatment in humans and with a time range from 1985 onwards. Data search was done between March and July 2020.

### 2.2. Selection Process

Articles on humans that met the following inclusion criteria were selected: Randomized Clinical Trials (RCTs) and systematic reviews of RCTs that used an intervention such as consumption of antioxidants, compared with any type of treatment or placebo, in a population with a confirmed diagnosis of COPD and evaluating the following outcomes: exacerbations (probability, number in a predetermined time, severity, and/or prevention), hospital readmissions related to respiratory symptoms, and changes in lung function (FEV_1_, FVC, FEV_1_/FVC, among others) evaluated by spirometry. The RCTs that were part of a systematic review identified during the search were excluded as primary studies. No formal assessment of the quality of the included studies was performed.

### 2.3. Information Synthesis

The synthesis of the results was carried out in a narrative way for each intervention (antioxidants) evaluated. As subgroups of interest, the categories of antioxidant effect according to disease severity and administered dose of the intervention were identified. In addition, the effect of antioxidants on the primary and secondary outcomes determined by the study authors was independently analyzed.

## 3. Results

**Figure 2:**
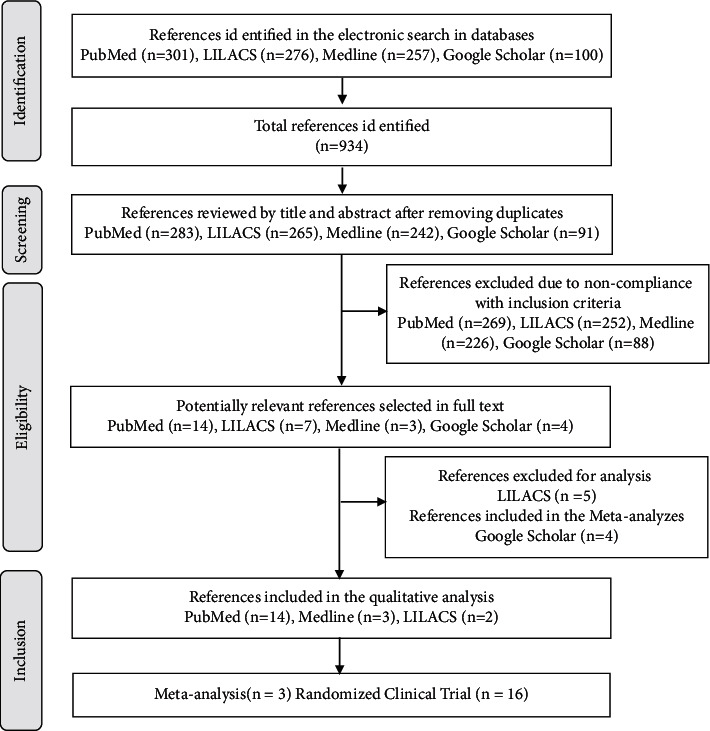
Flowchart for studies included in the scoping review.

19 studies, 3 meta-analyses and 16 RCTs, were selected after the reference selection process according to the inclusion criteria established for this review (Figure 2).

Table 1 shows the characteristics of the included studies in relation to the design, sample size, and age of the participants in each group, as well as the criteria used to define COPD in the primary studies (the definition in the secondary studies was not available). 25% of the articles (*n* = 4/16) used the criteria according to the Global Initiative for Chronic Obstructive Lung Disease (GOLD) 1, for the diagnosis of the disease when recruiting participants, while other studies were based on findings of lung function during spirometry or, less frequently, according to the criteria of the Medical Research Council (MRC) [29]. Likewise, the inclusion of patients with moderate-to-severe stages of COPD was more usual. The most frequently evaluated primary outcomes were changes in respiratory symptoms associated with exacerbation and effects on pulmonary function parameters; also, the effects of the intervention on biomarkers were reported, including oxidative stress and quality of life.

The results of each intervention are presented below in descending order, according to the frequency of use in the included studies:

1. N-Acetylcysteine. N-Acetylcysteine was evaluated in 42% of the articles (*n* = 8/19), being the most used intervention in the treatment of patients with COPD with doses of 600 mg, once or twice a day. Its effect was analyzed mainly on the probability of presenting an exacerbation, as well as its association with the amount or time to develop this type of clinical picture (Table 2). One of the secondary studies published in 2016 [30] reported a 15% decrease in the probability of developing an exacerbation during the observation period in subjects who took N-Acetylcysteine for more than six months. Likewise, a lower frequency of hospital admissions was found in the patients who received the intervention and fewer days in the duration of the exacerbation episode (Table 3). Along the same lines, Rogliani et al. [31] reported a greater preventive effect, both for the duration of the exacerbation and for the risk of being hospitalized for said episode, and another systematic review [32] even reported a relative benefit in reducing symptoms as well as for the absence of exacerbation for the patients who took N-Acetylcysteine in comparison with those who took placebo (48.5% versus 31.2%, respectively), finding a necessary number of 5.8 to treat (95% CI = 4.5–8.1). The ECCA developed by Tse et al. [33] found a difference in the probability of exacerbation per year with high doses (1200 mg/day), especially in the time for the development of the picture in patients categorized as high risk (C-D) (Table 4). On the other hand, the primary studies did not always present results in favor of N-Acetylcysteine. For example, Aytemur et al. [34] reported absence of difference in the score that classified the improvement of symptoms, in the time or number of exacerbations, in the time until hospitalizations or the number of them, and in the levels of arterial gases or lung function parameters. A similar scenario has been reported by Schermer et al. [35], in which, compared to placebo, patients in the N-Acetylcysteine group presented 1.35 times more exacerbations per year, in addition to similar scores between both groups on the scale of evaluation of CRQ quality of life and lung function parameters at three years. Likewise, Decramer et al. [36] and Parr et al. [37] did not find a superior effect of this molecule in terms of the number of exacerbations presented. According to the route of administration, the use of inhaled N-Acetylcysteine has been reported less frequently, although without changes in well-being symptoms, cough, dyspnea in the morning or with exercise, or the characteristics of expectoration [38].
2. Vitamin D. Vitamin D has been valued as a potential antioxidant in COPD patients at a dose of around 120,000 IU (Table 2); however, its effect has been contradictory. Martineau et al. [39] found no differences in time to moderate/severe exacerbation or upper respiratory infection between patients who consumed vitamin D and those who did not, as well as Jolliffe et al. [40] (1 in the probability of a moderate or severe exacerbation or changes in lung function parameters, Table 3). However, when stratifying the analysis according to baseline vitamin D levels, in those patients with levels below 50 nmol/L, a protective effect on exacerbations was evidenced (Table 4).
3. Vitamin E. Nadeem et al. [41] found that, at a dose of 800 IU, the FEV_1_ values were similar between those who received vitamin E supplementation and the comparison group, as well as the levels of oxidative stress markers and the indicators of antioxidant status (Table 3).
4. Other antioxidants. The findings on supplementation with Melatonin [42] showed a reduction in oxidative stress and improvement in signs such as dyspnea. In the case of Zinc [43], it was not possible to show an improvement in lung function parameters but in the levels of this metabolite and with those of superoxide dismutase (Table 3). From a nutritional perspective, patients with nutritional support during hospitalization or those who received a formula high in fat and low in carbohydrates [44, 45] presented less changes in lung function as well as in respiratory symptoms but without clear changes in gas arterials levels.
5. Nutraceuticals. Plants or fruits extracts have also been investigated, finding that *Chlorella vulgaris* [46] can induce significant changes in oxidative stress biomarkers (MDA, VIT E, VIT C, GSH, GPx, CAT, and SOD) as shown in Table 3. Supplementation with oligomeric proanthocyanidins [47], a nutraceutical product extracted from grape seeds, significantly improved the oxidative state of COPD patients, reducing the concentration of malondialdehyde (MDA) and the activity of superoxide dismutase (SOD); this was possibly related to the antioxidant effects of polyphenols present in grape seeds; in addition, it managed to improve the lipid profile of the patients. The pomegranate extract supplement [48], very rich in ellagitannin polyphenols, especially punicalagin and punicalin, did not show statistically significant differences in any of the parameters for bioavailability (biochemical and hematological) and for lung function (FEV_1_, FVC, FEV_1_/FVC, PaCO_2_, and PaO_2_), compared to the control group during the 5 weeks of follow-up. However, these results do not allow ruling out the possibility that longer supplementation periods (months or years) with nutraceuticals may have much more significant and beneficial effects in COPD patients. In fact, from a dietary point of view, a preventive (chronic) rather than a therapeutic (acute) effect should be expected for foods rich in dietary phytochemicals [49].

## 4. Discussion

This scoping review evaluated the effect of different antioxidant interventions for the prevention of exacerbations, improvement of respiratory symptoms, or changes in oxidative stress markers in patients diagnosed with COPD. In general, the beneficial or protective effects of the different types of antioxidants studied to date are not clear. Although the secondary studies included in this review reported a decrease in exacerbations with the use of N-Acetylcysteine, the differences found in the results of the outcomes between the groups compared were small or not clinically relevant, which prevents estimating significant associations. It is recommended that analyses in secondary studies be performed based on heterogeneity between primary studies (e.g., in dose or duration). The results were less clear in the supplementation interventions with vitamins and the other antioxidants.

The evaluation of the potential effects of antioxidants in COPD patients is a promising line of research, due to the rationale behind the pathophysiological mechanisms that cause oxidative stress in this disease and that may include development of recurrent inflammation, imbalance protease/antiprotease, and environmental toxins, as well as the genetic response of the host. Smoking is the main risk factor for COPD; it increases ROS in the lungs of exposed individuals; an inhaled cigarette smoke contains around 1015 free radicals (generation of hydrogen peroxide, superoxide dismutase, among others) in the gas phase. After said exposure, injury occurs at the level of the respiratory tract and a systemic response characterized by the decrease in antioxidant molecules. This oxidative stress triggers cellular and molecular reactions such as activation of the creatine kinase pathway and transcription factors, release of inflammatory mediators, cellular injury, and apoptosis [49, 50]. On the other hand, firewood is the most widely used biomass in the world and its fuel is widely used for cooking in developing countries.

In the Andean zone, COPD generated by wood smoke is an underestimated public health problem in terms of frequency and severity. It is postulated that its effect is that the smoke from this and other biomass contains a mixture of particles of variable size and chemical composition, called Particles of Matter (PM). Some of them, especially the fine and ultrafine ones, are breathed in as well as sequester iron from lung cells. When cells detect iron deficiency, they try to restore its availability by generating an increase in ROS production and a decrease in antioxidant mechanisms such as superoxide dismutase (SOD), glutathione (GSH), and ascorbic acid, promoting a state of inflammation pulmonary and systemic as well as oxidative imbalance with genotoxic consequences and cell damage [51].

The criteria used for the selection of the articles included in our review (*n* = 19) focused on the specific choice of in vivo studies of the ECCA type carried out in patients with COPD and that evaluated the effects of antioxidant consumption, specifically in this population. However, patients with other types of pathologies could also benefit from the effects of antioxidants, especially those from foods available in different countries, such as dry grains, red wine, a variety of fruits and vegetables, and coffee [20]. The latter is one of the products that contribute significantly to the dietary intake of antioxidants and is highly available in the Andean countries. Up to 23 different compounds have been found to be present in roasted coffee products, especially 5-caffeoylquinic acid, a type of chlorogenic acid, and antioxidant-caffeic acid in HT-29 cells (human colon) [52]. However, roasted coffee beans are not the only ones that provide compounds with high nutritional value. The so-called “coffee honey or mucilage,” the part of the fruit that covers the coffee almond, is considered one of the by-products with a large number of properties, high content of sugars, polyphenols, and chlorogenic acid, which make it raw material of interest for the industrial production of food supplements and energy drinks [53].

Currently, there are blends of black tea or amino acids with coffee mucilage concentrated on in the market, which turns them into nutraceutical foods that can be used as an antioxidant supplement (polyphenol content 7 times more than grapes), which contains phytonutrients that disarm free radicals of the body to maintain a healthier immune system. It should be noted that the list of foods with potentially antioxidant properties can be even bigger; products such as eucalyptus, moringa, noni, turmeric, garlic, rosemary, and lemon are widely used by specific communities that are in charge of popularizing them due to the beneficial effects observed clearly at an empirical and cultural level, in addition to being inexpensive and widely available foods or plants, but which have been little studied by experts at a scientific-experimental level.

Our scoping review shows that it has not been possible to define which antioxidant drug or nutraceutical, or a combination thereof, may be the best candidate at the level of in vivo research, despite the fact that in vitro studies of some products have reported potential beneficial findings [52, 54, 55]. The above situation could be explained by a failure of the antioxidant (or its vehicles) to be directed to the correct cell compartment, due to a possible low potency, insufficient doses, or short supplementation periods; therefore, ideally, a small molecule with good bioavailability and a broad antioxidant spectrum would be a better candidate [50]. Taking into account the above, it is essential to continue in the search for the best intervention that allows reducing oxidative stress through antioxidant substances, with an effect at a systemic level, and that counteracts the negative effects of the state produced by cigarettes and other triggers in COPD patients.

## 5. Conclusion

The most indicated antioxidants are N-Acetylcysteine, vitamins E and D, and Zinc. Other antioxidants from plants or fruits extracts are also being investigated. The beneficial effect of antioxidants in stable or exacerbated patients is not clear, but theoretical and biological arguments of benefit justify lines of research that specify the impact on reducing oxidative stress and negative effects in COPD.

## Figures and Tables

**Table 1:** Characteristics of the included studies.

ID	Author	Year	Design	Sample size		Age^*∗*^ (years)		Diagnostic criteria for COPD
				Intervened	Controls	Intervened	Controls	
1	Fowdar [28]	2016	Systematic review and meta-analysis	1339	1352	NR	NR	Does not apply
2	Aytemur [34]	2015	Prospective, double blind, controlled study	19	19	68.6 (7.5)	69.4 (9.9)	Spirometry, smoking history of at least 20 pack-years
3	Kirkil [43]	2008	RCT	15	15	61.06 (2.89)	62.26 (3.19)	Medical history: history of smoking and irreversible airway obstruction with <15% change in baseline FEV1 in response to b2 agonists
4	Tse [33]	2014	RCT	52	56	70.9 (1.7)	70.7 (1.4)	Spirometry: postbronchodilator FEV_1_/FVC <0.70
5	Nadeem [41]	2008	RCT	10	14	60.10 (1.16)	54.86 (7.13)	GOLD criteria
6	Decramer [36]	2005	RCT phase III, double blind	256	267	62	62	COPD related to smoking
7	Schermer [35]	2009	RCT	190	96	58.8	59.6	Chronic bronchitis or mild-to-moderate COPD. It does not report how the diagnosis was confirmed
8	Cai [45]	2003	RCT	30	30	62 (7)	63 (6)	Patients with confirmed COPD, with elevated arterial carbon dioxide
9	Stey [32]	2000	Systematic review	996	1015	NR	NR	Does not apply
10	Martineau [39]	2015	RCT double blind, placebo controlled	122	118	64.8 (7.9)	64.5 (9.2)	History of COPD, emphysema, or chronic bronchitis from medical history
11	Vermeeren [44]	2004	RCT double blind, placebo controlled	23	24	66 (8)	65 (10)	GOLD criteria
12	Dueholm [38]	1992	RCT double blind	31	34	52.7 (1.76)	53.9 (1.88)	MRC criteria
13	Parr [37]	1987	RCT	258	268	63.3	62.7	MRC criteria
14	Jolliffe [40]	2018	Systematic review and meta-analysis	381	398	65.0 (40–86)^∗∗^	65.0 (40–86)^∗∗^	Does not apply
15	Rogliani [31]	2019	Systematic review and meta-analysis	2615	1151	(65–73)	(65–73)	Does not apply
16	Meng-Chun [47]	2018	RCT, double blind	13	14	71 (2)	71 (2)	Does not report how the diagnosis was confirmed
17	Panahi [46]	2012	RCT, open label	28	29	M: 50.5(3.2)	M: 52.8 (2.8)	It does not report how the diagnosis was confirmed
						F: 48.1 (2.9)	F: 51.6 (3.8)	
18	Matos [42]	2012	RCT, double blind, placebo controlled	18	18	68.22 (6.8)	64.94 (8.5)	GOLD criteria
19	Cerdá [48]	2006	RCT, double blind, placebo controlled	15	15	60 (10.9)	63.40 (8.9)	COPD diagnosed by a doctor

^*∗*^ Age reported as mean and standard deviations; NR : not reported; ^∗∗^Age reported as median and interquartile range; M : Male; F : Female.

**Table 2:** Type of intervention and primary outcomes evaluated.

ID	Author	Intervention name	Time (months)	Dose (mg/day)	Via	Main outcome	Intervention	Comparison	*P* value
1	Fowdar [28]	NAC	<6 versus 6	>600 versus 600	Oral	Exacerbation: acute worsening of respiratory symptoms resulting in the requirement for further therapy, triggered by bacterial or viral respiratory infection, environmental contaminants, or unknown factors	^*∗*^0.85 (0.76–0.96)		0.006
2	Aytemur [34]	NAC	1	200	Oral	Symptom improvement: 7-point scale (1 is the worst symptom and 7 is the best state)	0.62	0.31	0.96
3	Kirkil [43]	Zinc picolinate	2	22	Oral	Pulmonary function parameters:			
						(i) FEV_1_ (%)	42.73 (5.83)	42.93 (2.71)	0.89
						(ii) FEV_1_/FVC (%)	64.26 (7.50)	64.00 (3.94)	0.87

4	Tse [33]	NAC	12	1200	Oral	Exacerbation: presence of two of the following symptoms: increased shortness of breath, increased volume of sputum, and increased purulence of sputum	0.96/year	1.71/year	0.019
5	Nadeem [41]	Vitamin E	2	800 UI	Oral	Pulmonary function parameters:	51.56 (18.84)	47.14 (20.56)	NR
						(i) FEV_1_			

6	Decramer [36]	NAC	36	600	Oral	Annual reduction in FEV_1_ (ml)	54 (6)^∗∗^	47 (6)^∗∗^	NR
7	Schermer [35]	NAC	36	600	Oral	Annual exacerbation rate	Rate ratio		
						(i) Intention to treat	1.35		0.054
						(ii) Per protocol	1.58		0.099

8	Cai [45]	High-fat, low-carb formula	3 weeks	237 mL	Oral	Changes in pulmonary function:	48 (9)	42 (9)	<0.05
						(i) FEV_1_			

9	Stey [32]	NAC	1 a 6	600	Oral	Exacerbation prevention	^*∗*^1.56 (1.37–1.77)	NR	
10	Martineau [39]	Vitamin D	12	3 (120.000 UI)	Oral	Time to moderate or severe exacerbation	+0.86 (0.60–1.24)	0.42	
11	Vermeeren [44]	Nutritional support	8 days	125 ml (20% protein, 20% fat, 60% energy)	Oral	Change in lung function parameters:			
						(i) FEV_1_ (%)	5 (6)	5 (9)	<0.05
						(ii) IVC (%)	7 (12)	10 (17)	<0.05

12	Dueholm [38]	NAC	1	8	Inhaled	Changes in symptoms (score of 0–10)			NR
						(i) Wellness	0.16 (−1.03; 0.75)		
						(ii) Cough	1.05 (−0.12; 1.36)		
						(iii) Dyspnea in the morning	0.49 (−0.16; 1.19)		
						(iv) Dyspnea with exercise	0.55 (−0.09; 1.39)		
						(v) Booger	0.50 (−0.29; 1.11)		
						Expectoration:			
						(i) Quantity	0.15 (−0.39; 0.93)		
						(ii) Viscosity	0.14 (−0.77, 0.64)		
						(iii) Difficulty	0.23 (−1.18, 0.88)		

13	Parr [37]	NAC	12	200	Oral	Number of exacerbations (patients/year)	2.2	2.5	NR
14	Jolliffe [40]	Vitamin D	.	.	Oral	Moderate or severe exacerbation	0.94 (IC95% 0.78; 1.13)	0.52	
15	Rogliani [31]	Erdosteine,	6 ECA: 13	E: 600	Oral	Acute exacerbation:			<0.001
		NAC	1 ECA: 8	C: 1500		(i) General	0.61 (0.51–0.82)^*∗*^		
		Carbocysteine		N: 1200		(ii) Erdosteine,	0.76 (0.66–0.87)^*∗*^		
						(iii) NAC	0.68 (0.49–0.93)^*∗*^		
						(iv) Carbocysteine	0.44 (0.24–0.82)^*∗*^		

16	Meng-Chun [47]	Oligomeric proanthocyanidins	2	150	Oral	Pulmonary function parameters:			NR
						(i) FEV_1_ (%)	65.85 (7.97)	69.71 (6.38)	
						(ii) FVC (%)	43.92 (5.39)	46.29 (4.56)	
						(iii) FEV_1_/FVC (%)	53.54 (4.79)	52.79 (4.43)	
						(iv) PEFR (%)	45.54 (4.49)	45.93 (4.12)	

17	Panahi [46]	*Chlorella vulgaris* extract	2	2700	Oral	Magnitude of change in oxidative stress biomarkers:			
						(i) MDA	−2.03 (0.86)	−0.60 (1.47)	0.025
						(ii) VIT E	2.78 (15.88)	0.05 (0.25)	<0.001
						(iii) VIT C	0.46 (0.34)	−0.04 (0.20)	<0.001
						(iv) GSH	9.37 (2.34)	2.60 (5.20)	<0.001
						(v) GPx	2.26 (0.44)	0.15 (1.12)	<0.001
						(Vi) CAT	10.51 (5.01)	−1.73 (5.26)	<0.001
						(vii) SOD	1.01 (0.56)	0.60 (0.78)	0.011
						(viii) TAL	0.30 (0.24)	0.20 (0.29)	0.104

18	Matos [42]	Melatonin	3	3	Oral	Oxidative lung stress: isoprostane-8 (average difference)	7.708	−0.613	NR
19	Cerdá [48]	Granada juice	1.2	400 ml	Oral	Pulmonary function parameters:			NR
						(i) FEV_1_	1.38 (0.61)	0.91 (0.92)	
						(ii) FVC	2.94 (0.76)	2.03 (0.39)	
						(iii) FEV_1_/FVC	45.60 (13.28)	44.1 (0.44)	
						(iv) Pa0_2_	72.19 (8.70)	71.67 (0.97)	
						(v) PC0_2_	40.35 (4.66)	42.50 (0.95)	

NAC : N-Acetylcysteine; ^*∗*^ relative risk (95% CI); +hazard ratio (95% CI); ^∗∗^ average difference (standard error); rate ratio: number of times per year that patients treated with NAC have exacerbations compared to placebo; MDA: malondialdehyde; vit E: vitamin E; vit C: vitamin C; GSH: glutathione; GPX: glutathione peroxidase; CAT: catalase; SOD: superoxide dismutase; TAS: total antioxidant status.

**Table 3:** Type of intervention and secondary outcomes evaluated.

ID	Author	Intervention	Secondary outcomes	Intervention	Comparison	*P* value
1	Fowdar [28]	NAC	Hospital admissions (absolute frequency): 3 studies	37/50	55/50	NR
				26/52	45/56	
				33/482	36/482	
			Duration of exacerbation (days): 2 studies	17.2	23.7	NR
				14.8	19.2	<0.0001

2	Aytemur [34]	NAC	Arterial Gases:			
			(i) Pa02 (mmHg)	70.5 (15.0)	68.7 (12.4)	0.777
			(ii) PaC02 (mmHg)	43.5 (9.1)	44.5 (5.3)	NR
			(iii) Sa02 (%)	92.9 (4.5)	92.6 (4.5)	0.979
			FEV_1_ (ml)	1239 (543)	1180 (535)	0.076
			Hospital stay (days)	10.5 (3.8)	9.8 (3.0)	0.52
			Number of exacerbations at 6 months	1.1 (1.3)	1.0 (1.4)	0.788
			Time to exacerbation (days)	50.4 (66.2)	22.7 (27.6)	0.347
			Number of hospitalizations at six months of follow-up	0.9 (1.1)	0.6 (0.9)	0.424
			Time to hospital admission (days)	45.6 (67.2)	24.6 (41.5)	0.373

3	Kirkil [43]	Zinc	Serum levels: baseline versus postsupplementation			
			(i) MDA (nmol/mL)	0.51 (0.15)	0.46 (0.38)	0.061
			(ii) SOD (U/mL)	0.16 (0.02)	0.18 (0.01)	0.029
			(iii) CAT (k/mL)	14.79 (3.03)	16.27 (1.10)	0.523
			(iv) Zinc (mg/dL)	77.33 (4.29)	87.36 (4.96)	<0.001

4	Tse [33]	NAC	Time to exacerbation (days)	261.5 (18.4)	239.5 (17.9)	0.17
			Exacerbation-free patients	53.8%	37.5%	0.088

5	Nadeem [41]	Vitamin E	Indicators of Oxidative Stress:			
			(i) Generation of superoxide leukocytes (nm O2¯ produced/15 min/2.5 × 106 cells)	16.54 (7.77)	14.6 (8.53)	NR
			(ii) Plasma peroxidized lipids (*μ*M/l)	3.79 (0.94)	3.93 (0.99)	
			(iii) Total plasma protein carbonyls (nm/mg protein)	1.14 (0.26)	1.16 (0.25)	
			(iv) Total plasma proteins sulfhydryls (nm/l)	0.53 (0.09)	0.51 (0.08)	
			(v) Total nitrates and nitrites (*μ*M/l)	69.09 (35.51)	68.8 (39.64)	
			Antioxidant status indicators:			NR
			(i) Red cell superoxide dismutase (U/g Hb)	2040.4 (783.7)	2126.1 (831.4)	
			(ii) Red cell catalase (U/g Hb)	3382.1 (1012.4)	3005.1 (1062.9)	
			(iii) Total blood glutathione (nM)	1.21 (0.65)	0.78 (0.57)	
			(iv) Red cell glutathione peroxidase (*μ*M oxidized NADPH/min/g Hb)	50.6 (13.5)	47.3 (13.2)	
			(v) Plasma glutathione peroxidase (oxidized nM NADPH/min/ml)	169.7 (73.8)	184.1 (55.8)	
			(vi) Total plasma antioxidant power (*μ*M)	554.2 (48)	650.01 (183.3)	

6	Decramer [36]	NAC	Probability of exacerbations per year	0.99^*∗*^ (0.89–1.10)		0.85
			Probability of exacerbations per year in patients without inhaled corticosteroids	0.79^*∗*^ (0.63–0.98)		0.04
			EuroQoL-5D (first year)	0·018 (–0·015; 0·05)	0·037 (0·004; 0·069)	0.414
			EuroQoL-5D (after the first year)	–0·02 (–0·034; 0·005)	–0·02 (–0·034; –0·004)	0.965
			St. George's respiratory questionnaire (1st year)	–3.76 (–5.55; –1.98)	–4.95 (–6.74; –3.16)	0.358

			St. George's respiratory questionnaire (annual decrease)	1.45 (0.52; 2.38)	1.24 (0.28; 2.21)	0.763

7	Schermer [35]	NAC	Quality of life (total score)			
			(i) Score chronic respiratory questionnaire CRQ	<0.5	<0.5	0.306
			Pulmonary function parameters (at three years):		−60 (5.4)^∗∗^	NR
			(i) FEV_1_ (mL) after BD	−64 (5.4)^∗∗^	−62 (7.1)^∗∗^	
			(ii) Tight	−60 (7.5)^∗∗^		
			(iii) Per protocol		−65 (9.4)^∗∗^	
			(iv) FVC (mL) after BD	−79 (9.5)^∗∗^	−57 (13.1)^∗∗^	
			(v) Tight	−51 (13.9)^∗∗^		

8	Cai [45]	High-fat, low-carb formula	Arterial gases:			NR
			(i) PH	7.38 (0.21)	7.37 (0.21)	
			(ii) Pc0_2_ (mm Hg)	42 (3)	45 (3)	
			(iii) Po_2_ (mm Hg)	79 (4)	71 (6)	

9	Stey [32]	NAC	Decrease in symptoms (cough, mucopurulent sputum, and dyspnea)	1.78^*∗*^ (1.54–2.05)	NR	
10	Martineau [39]	Vitamin D	Time it takes for the first upper respiratory infection to appear	0.95^*∗*^ (0.69–1.31)	0.75	
11	Vermeeren [44]	Nutritional support	Changes in symptoms (score):			
			(i) Dyspnea at rest	−3.8 (2.5)	−4.3 (3.2)	<0.05
			(ii) Dyspnea while eating	−4.2 (2.5)	−4.7 (3.3)	<0.05
			(iii) Loss of appetite	−2.7 (3.2)	−2.6 (2.9)	<0.05
			(iv) Early satiety	−3.2 (3.3)	−2.5 (3.3)	<0.05
			(v) Borborygmus	−2.4 (3.4)	−3.0 (2.8)	<0.05
			(vi) Fatigue	−4.2 (3.7)	−3.9 (3.5)	<0.05

12	Dueholm [38]	NAC	Pulmonary function:			
			(i) FEV_1_	1.9 (0.18)	2.0 (0.13)	NS
			(ii) FVC	3.0 (0.21)	2.9 (0.18)	
			(iii) PEFR	356.7 (29.64)	354.6 (25.07)	
			Number of exacerbations	9	7	NR

14	Jolliffe [40]	Vitamin D	Time to first exacerbation (3 studies)	0.93^*∗*^ (0.74–1.17)		0.53
			% FEV_1_ at the end of the study (3 studies)	54.9 (21.0)	54.9 (21.9)	0.16
			% HRV at the end of the study (3 studies)	88.8 (20.0)	89.9 (21.8)	0.49

15	Rogliani [31]	Erdosteine	Risk of at least one exacerbation			NR
			(i) General	0.90^*∗*^ (0.83–0.96)		
			(ii) Erdosteine	0.82^*∗*^ (0.70–0.95)		
			(iii) NAC	0.88^*∗*^ (0.74–1.05)		
			(iv) Carbocysteine	0.94^*∗*^ (0.83–1.07)		
		NAC	Duration of exacerbation (days)			NR
			(i) General	−2.63 (−4.03; –1.23)		
			(ii) Erdosteine	−2.04 (−3.51; –0.57)		
			(iii) NAC	−3.56 (−5.46; –1.66)		
		Carbocysteine	Risk of hospitalization due to exacerbation			NR
			(i) General	0.69^*∗*^ (0.52–0.91)		
			(ii) Erdosteine	0.56^*∗*^ (0.33–0.94)		
			(iii) NAC	0.75^*∗*^ (0.51–1.10)		

16	Meng-Chun [47]	Oligomeric proanthocyanidins	Plasma concentration of oxidative markers: MDA (nmol/ml)	5.87 (0.27)	7.95 (0.41)	NR
			(i) SOD (U/g protein)	2.70 (0.45)	5.37 (0.87)	
			(ii) Catalase (U/g protein)	13.42 (1.25)	16.51 (1.65)	
			(iii) GSH–PX (U/g protein)	0.14 (0.01)	0.19 (0.03)	
			Plasma lipids:			NR
			(i) Total cholesterol (mg/dl)	198 (13)	202 (9)	
			(ii) Triglycerides (mg/dl)	114 (20)	125 (21)	
			(iii) HDL (mg/dl)	59 (4)	47 (4)	
			(iv) LDL (mg/dl)	120 (12)	126 (7)	

18	Matos [42]	Melatonin	IL-8 (pg/mL)	2.24 (0.98)	2.41 (0.82)	NR
			FVC (L)	2.76 (0.67)	2.90 (0.70)	
			FEV_1_ (L)	1.46 (0.54)	1.44 (0.46)	
			MRC score	1.56 (1.38)	1.56 (1.38)	
			6-MWT (m)	391 (56)	389 (76)	

NAC : N-Acetylcysteine; ^*∗*^ hazard ratio (95% CI); ^∗∗^ average (standard error); IVC : inspiratory vital capacity; GSH-Px : glutathione peroxidase; FVC : forced vital capacity; 6-MWT: 6-minute walk test. ^∗∗^ Measurements at the time of hospital discharge. NS : not significant; NR : no report; MDA : plasma malondialdehyde; SOD : superoxide dismutase; CAT : catalase.

**Table 4:** Outcomes by subgroups of patients.

ID	Author	Intervention	Subgroup	Outcome	Intervention	Comparison	*P* Value
1	Fowdar [28]	NAC	Dose	Exacerbation			NR
			(i) 600 mg/day		0.90 (0.82–1.00)		
			(ii) <600 mg/day		0.83 (0.69–0.99)		
			Use time				NR
			(i) >6 months		0.85 (0.74–0.98)		
			(ii) <6 months		0.81 (0.54–1.21)		

4	Tse [33]	NAC	High risk of exacerbation:	Time to exacerbation	258.2 (20.8)	203.6 (20.4)	0.02
			(i) Categories C-D				
			Low risk of exacerbation:		272.1 (39.0)	337 (21.3)	0.34
			(i) Categories A-B				
			High risk of exacerbation:	Amount of hospital admissions			
			(i) Categories C-D				
			(ii) 4 months		0.31 (0.1)	0.29 (0.09)	0.91
			(iii) 8 months		0.49 (0.14)	0.56 (0.13)	0.69
			(iv) 12 months		0.51 (0.14)	0.93 (0.18)	0.079
			Low risk of exacerbation:				
			(i) Categories A-B				
			(ii) 4 months		0.08 (0.08)	0.07 (0.07)	0.9
			(iii) 8 months		0.23 (0.12)	0.13 (0.13)	0.6
			(iv) 12 months		0.31 (0.17)	0.13 (0.13)	0.43
			FEV_1_:	Exacerbation frequency	1.21 (0.25)	2.3 (0.36)	0.16
			(i) <50%				

10	Martineau [39]	Vitamin D	Levels	Time to moderate or severe exacerbation			
			(i) <50 nmol/L		0.57		0.021
			(ii) 50 nmol/L		1.45		0.21
			(iii) or more				

14	Jolliffe [40]	Vitamin D	Levels	Incidence of moderate or severe exacerbation			
			(i) <25 nmol/L			0.55 (CI 95%: 0.36; 0.84)^∗∗^	0.006
			(ii) ≥25 nmol/L			1.04 (CI 95%: 0.85; 1.27)^∗∗^	0.71
			Levels	Incidence of moderate or severe exacerbation			
			(i) 25.0–49.9 nmol/L			1.00 (CI 95%: 0.77; 1.31)^∗∗^	0.97
			(ii) 50.0–74.9 nmol/L			1.06 (CI 95%: 0.70; 1.61)^∗∗^	0.78
			(iii) ≥75 nmol/L			1.17 (CI 95%: 0.73; 1.87)^∗∗^	0.52

NAC : N-Acetylcysteine; ^*∗*^ relative risk (95% CI); ^∗∗^ aIRR: adjusted incidence rate ratio.

## Data Availability

The data are available from the corresponding author upon request.

## References

[1] Global Initiative for Chronic Obstructive Lung Disease (GOLD) (2017). *Guía de bolsillo para el diagnóstico, manejo y prevención de la EPOC: una guía para profesionales de la asistencia sanitaria*.

[2] GBD 2017 Disease and Injury Incidence and Prevalence Collaborators (2018). Global, regional, and national incidence, prevalence, and years lived with disability for 354 diseases and injuries for 195 countries and territories, 1990–2017: a systematic analysis for the Global Burden of Disease Study 2017. *Lancet*.

[3] GBD 2017 Disease and Injury Incidence and Prevalence Collaborators (2018). Global, regional, and national age-sex-specific mortality for 282 causes of death in 195 countries and territories, 1980–2017: a systematic analysis for the Global Burden of Disease Study 2017. *Lancet*.

[4] World Heatlh Organization (WHO) (2008). *World Health Statistics 2008*.

[5] Ministerio de Salud Y Protección Social, D. Administrativo de Ciencia, Tecnología e Innovación-Colciencias (2014). *Guía de práctica clínica Basada en la evidencia para la prevención, diagnóstico, tratamiento y seguimiento de la enfermedad pulmonar obstructiva crónica (EPOC) en población adulta*.

[6] Alcolea Batres S., Villamor León J., Álvarez-Sala R. (2007). EPOC y estado nutricional. *Archivos de Bronconeumología*.

[7] Hsieh M.-J., Yang T.-M., Tsai Y.-H. (2016). Nutritional supplementation in patients with chronic obstructive pulmonary disease. *Journal of the Formosan Medical Association*.

[8] Cao C., Wang R., Wang J., Bunjhoo H., Xu Y., Xiong W. (2012). Body mass Index and mortality in chronic obstructive pulmonary disease: a meta-analysis. *PLoS One*.

[9] American Thoracic Society (1999). Skeletal muscle dysfunction in chronic obstructive pulmonary disease: a statement of the American Thoracic Society and European Respiratory Society. *American Journal of Respiratory and Critical Care Medicine*.

[10] Orozco-Levi M., Lloreta J., Minguella J., Serrano S., Broquetas J. M., Gea J. (2001). Injury of the human diaphragm associated with exertion and chronic obstructive pulmonary disease. *American Journal of Respiratory and Critical Care Medicine*.

[11] Gea J., Casadevall C., Pascual S., Orozco-Levi M., Barreiro E. (2012). Respiratory diseases and muscle dysfunction. *Expert Review of Respiratory Medicine*.

[12] Orozco-Levi M., Coronell C., Ramírez-Sarmiento A. (2012). Injury of peripheral muscles in smokers with chronic obstructive pulmonary disease. *Ultrastructural Pathology*.

[13] Gea J., Martínez-Llorens J., Barreiro E. (2014). Alteraciones nutricionales en la enfermedad pulmonar obstructiva crónica. *Medicina Clínica*.

[14] Gea J., Estirado C., Barreiro E. (2017). Alterations in nutritional status and body composition in COPD patients. *BRN Rev*.

[15] Gea J., Sancho-Muñoz A., Chalela R. (2018). Nutritional status and muscle dysfunction in chronic respiratory diseases: stable phase versus acute exacerbations. *Journal of Thoracic Disease*.

[16] Sharma A., Tewari D., Nabavi S. F., Nabavi S. M., Habtemariam S. (2021). Reactive oxygen species modulators in pulmonary medicine. *Current Opinion in Pharmacology*.

[17] Swallow E. B., Reyes D., Hopkinson N. S. (2007). Quadriceps strength predicts mortality in patients with moderate to severe chronic obstructive pulmonary disease. *Thorax*.

[18] Jaitovich A., Barreiro E. (2018). Skeletal muscle dysfunction in chronic obstructive pulmonary disease. what we know and can do for our patients. *American Journal of Respiratory and Critical Care Medicine*.

[19] Engelen M. P., Rutten E. P., de Castro C. L., Wouters E. F., Schols A. M., Deutz N. E. (2007). Supplementation of soy protein with branched-chain amino acids alters protein metabolism in healthy elderly and even more in patients with chronic obstructive pulmonary disease. *American Journal of Clinical Nutrition*.

[20] Neha K., Haider M. R., Pathak A., Yar M. S. (2019). Medicinal prospects of antioxidants: a review. *European Journal of Medicinal Chemistry*.

[21] Román M., Alva A., Pinzón A., Carvajal K. G. (2016). Papel inmunomodulador y antioxidante del zinc y el selenio en el tratamiento coadyuvante de infecciones respiratorias graves. *Revista de Educación Bioquímica*.

[22] Díez J. M., Aranda S., Lesmes I. (2018). Recomendaciones dietéticas y suplementos nutricionales en la EPOC. *Revista de Patología Respiratoria*.

[23] Janssens W., Bouillon R., Claes B. (2010). Vitamin D deficiency is highly prevalent in COPD and correlates with variants in the vitamin D-binding gene. *Thorax*.

[24] Cuevas-schacht F., González-Enriquez H. (2004). Erdosteína como agente mucorregulador. *Neumologia y Cirugia de Torax*.

[25] Gillissen A. (2011). Grundlagen der antiinflammatorischen Wirkung von N-Acetylcystein und dessen therapeutische Einsatzmöglichkeiten. *Pneumologie*.

[26] European Nutraceutical Association (2021). Health, wellness and fitness.

[27] Scoditti E., Massaro M., Garbarino S., Toraldo D. M. (2019). Role of diet in chronic obstructive pulmonary disease prevention and treatment. *Nutrients*.

[28] Grant M. J., Booth A. (2009). A typology of reviews: an analysis of 14 review types and associated methodologies. *Health Information & Libraries Journal*.

[29] Fletcher C. M. (1960). Standardized questionaries on respiratory symptoms. *BMJ*.

[30] Fowdar K., Chen H., He Z. (2017). The effect of N-acetylcysteine on exacerbations of chronic obstructive pulmonary disease: a meta-analysis and systematic review. *Heart & Lung*.

[31] Rogliani P., Matera M. G., Page C., Puxeddu E., Cazzola M., Calzetta L. (2019). Efficacy and safety profile of mucolytic/antioxidant agents in chronic obstructive pulmonary disease: a comparative analysis across erdosteine, carbocysteine, and N-acetylcysteine. *Respiratory Research*.

[32] Stey C., Steurer J., Bachmann S., Medici T. C., Tramèr M. R. (2000). The effect of oral N-acetylcysteine in chronic bronchitis: a quantitative systematic review. *European Respiratory Journal*.

[33] Tse H. N., Raiteri L., Wong K. Y., Ng L. Y., Yee K. S., Tseng C. Z. S. (2014). Benefits of high-dose N-acetylcysteine to exacerbation-prone patients with COPD. *Chest*.

[34] Aytemur Z., Baysak A., Ozdemir O., Kose T., Abdullah S. (2015). N-acetylcysteine in patients with COPD exacerbations associated with increased sputum. *Wien Klinische Wochenschrift*.

[35] Schermer T., Chavannes N., Dekhuijzen R. (2009). Fluticasone and N-acetylcysteine in primary care patients with COPD or chronic bronchitis. *Respiratory Medicine*.

[36] Decramer M., Rutten-van Mölken M., Dekhuijzen P. R. (2005). Effects of N-acetylcysteine on outcomes in chronic obstructive pulmonary disease (Bronchitis Randomized on NAC Cost-Utility Study, BRONCUS): a randomised placebo-controlled trial. *The Lancet*.

[37] Parr G. D., Huitson A. (1987). Oral fabrol (oral N-acetylcysteine) in chronic bronchitis. *British Journal of Diseases of the Chest*.

[38] Dueholm M., Nielsen C., Thorshauge H. (1992). N-acetylcysteine by metered dose inhaler in thetreatment of chronic bronchitis: a multi-centre study. *Respiratory Medicine*.

[39] Martineau A. R., James W. Y., Hooper R. L. (2015). Vitamin D 3 supplementation in patients with chronic obstructive pulmonary disease (ViDiCO): a multicentre, double-blind, randomised controlled trial. *The Lancet Respiratory Medicine*.

[40] Jolliffe D. A., Greenberg L., Hooper R. L. (2019). Vitamin D to prevent exacerbations of COPD: systematic review and meta-analysis of individual participant data from randomised controlled trials. *Thorax*.

[41] Nadeem A., Raj H. G., Chhabra S. K. (2008). Effect of vitamin E supplementation with standard treatment on oxidant-antioxidant status in chronic obstructive pulmonary disease. *Indian Journal of Medical Research*.

[42] De Matos Cavalcante A. G., De Bruin P. F. C., De Bruin V. M. S. (2012). Melatonin reduces lung oxidative stress in patients with chronic obstructive pulmonary disease: a randomized, double-blind, placebo-controlled study. *Journal of Pineal Research*.

[43] Kırkıl G., Hamdi Muz M., Seçkin D., Şahin K., Küçük Ö. (2008). Antioxidant effect of zinc picolinate in patients with chronic obstructive pulmonary disease. *Respiratory Medicine*.

[44] Vermeeren M., Wouters E. F., Geraerts-keeris A. J., Schols A. M. (2004). Nutritional support in patients with chronic obstructive pulmonary disease during hospitalization for an acute exacerbation; a randomized controlled feasibility trial. *Clinical Nutrition*.

[45] Cai B., Zhu Y., Ma Y. i. (2003). Effect of supplementing a high-fat, low-carbohydrate enteral formula in COPD patients. *Nutrition*.

[46] Panahi Y., Tavana S., Sahebkar A., Masoudi H., Madanchi N. (2012). Impact of adjunctive therapy with Chlorella vulgaris extract on antioxidant status, pulmonary function and clinical symptoms of patients with obstructive pulmonary diseases. *Scientia Pharmaceutica*.

[47] Lu M.-C., Yang M.-D., Li P.-C. (2018). Effect of oligomeric proanthocyanidin on the antioxidant status and lung function of patients with chronic obstructive pulmonary disease. *In Vivo*.

[48] Cerdá B., Soto C., Albaladejo M. D. (2006). Pomegranate juice supplementation in chronic obstructive pulmonary disease: a 5-week randomized, double-blind, placebo-controlled trial. *European Journal of Clinical Nutrition*.

[49] Fischer B., Voynow J., Ghio A. (2015). COPD: balancing oxidants and antioxidants. *International Journal of Chronic Obstructive Pulmonary Disease*.

[50] Kirkham P. A., Barnes P. J. (2013). Oxidative stress in COPD. *Chest*.

[51] Silva R., Oyarzún M., Olloquequi J. (2015). Mecanismos patogénicos en la enfermedad pulmonar obstructiva crónica causada por exposición a humo de biomasa. *Archivos de Bronconeumología*.

[52] Bakuradze T., Lang R., Hofmann T. (2010). Antioxidant effectiveness of coffee extracts and selected constituents in cell-free systems and human colon cell lines. *Molecular Nutrition & Food Research*.

[53] Puerta-Quintero G. I., Ríos-Arias S. (2011). Composición química del mucílago de café, según el tiempo de fermentación y refrigeración. *CENICAFE*.

[54] Rao N. Z., Fuller M. (2018). Acidity and antioxidant activity of cold brew coffee. *Scientific Reports*.

[55] Liang N., Kitts D. (2014). Antioxidant property of coffee components: assessment of methods that define mechanisms of action. *Molecules*.

